# The impact of the face-to-face consultation on decisional conflict in complex decision-making in multiple sclerosis: A pilot study

**DOI:** 10.1177/2055217320959802

**Published:** 2020-10-20

**Authors:** David Daniel Wilkie, Alessandra Solari, Richard St. John Nicholas

**Affiliations:** Centre for Neuroscience, Faculty of Medicine, Imperial College London, London, UK; Imperial College Healthcare NHS Trust, Charing Cross Hospital, Fulham, UK; Unit of Neuroepidemiology, Fondazione IRCCS Istituto Neurologico Carlo Besta, Milan, Italy; Centre for Neuroscience, Faculty of Medicine, Imperial College London, London, UK; Imperial College Healthcare NHS Trust, Charing Cross Hospital, Fulham, UK; Centre for Neuroinflammation and Neurodegeneration, Faculty of Medicine, 4615 Imperial College London, London, UK

**Keywords:** Multiple sclerosis, decision-making, shared decision making, disease modifying therapies, patient engagement

## Abstract

**Background:**

The role of face-to-face consultations in medicine is increasingly being challenged. Disease activity, national guidelines, life goals e.g. pregnancy, multiple therapies and side effects need to be considered on starting disease modifying treatments (DMTs) in people with multiple sclerosis (pwMS).

**Objectives:**

We studied the impact of a face-to-face consultation on decision making, using decisional conflict (DC) as the primary outcome.

**Methods:**

Prospective cohort study of 73 pwMS attending clinics who were making decisions about DMTs followed for one year. Prerequisites and consultation features were measured with the SURE scale for DC used as the primary outcome at baseline and at one year.

**Results:**

The patient activation measure (PAM) was the only driver prior to the consultation associated with DC (p = 0.02) showing those less engaged were more likely to have DC. Overall, 51/73 (70%) of people made their treatment decision or reinforced a former decision during the consultation. We found making a treatment decision between the original consultation and the follow-up was associated with resolving DC (p = 0.008).

**Conclusions:**

Patient engagement impacts DC but the HCP delivering the optimal Shared Decision Making (SDM) approach is additionally significant in reducing DC. In complex decisions there is a clear role for face-to-face consultations in current practice.

## Background

The role of the face-to-face consultation, in terms of decisions about care, is being challenged. Historically, the healthcare professional (HCP) would lead on decision-making^1^ but today, with the aid of the Internet, patients can enter a consultation armed with both preferences and knowledge. Emphasis on self-management in chronic disease and the emergence of the ‘expert patient’ have questioned the utility of the ‘expert’ consultation.^2^ Furthermore, the elements driving a successful and satisfying consultation ultimately leading to a successful decision are opaque, thus how to harness its potential as the healthcare environment becomes ever more complex, is essential to its continuation.

Healthcare is full of complex decisions that patients and HCPs have to make; here we have focused on the decision people with Multiple Sclerosis (pwMS) face deciding about their treatment. No defined approach exists for choosing the right disease-modifying treatment (DMT) among many options for pwMS. There are now guidelines that recommend treatment early in the disease course^3^ and the question of starting treatment is a complex one, as the patient involved may have absent (or minimal) symptoms and other life priorities e.g. starting a family, requiring careful consideration.^4^ DMTs come with a wide range of benefits, routes of administration but also risk. This is further complicated by an increasing spectrum of therapeutic options with limited knowledge of relative efficacy or how they interact.^5^

There are three stages of the decision making process. Prerequisites e.g. what the patients bring to the consultation, including personality, role preference, mood, readiness to make a decision, and disease and risk knowledge; the process itself e.g. the patient/HCP interaction, best exemplified by Shared Decision Making (SDM), where the HCP and the patient share responsibility for agreeing a way forward. SDM allows people to be supported in understanding their medical condition, the treatment and support options available, whilst evaluating the risks and benefits of each option. SDM can also elicit a decision about a preferred course of action.^6^ Finally the consultation outcome is key and aims to resolve DC.^7^ Outcomes aim to assess the person’s satisfaction with a decision as opposed to the impact on their condition and how this evolves over time. Outcomes such as the Decisional Conflict (DC) scale measure a person’s perceived uncertainty about a decision to be made as a continuum, alternatively this can be measured using a binary outcome using the SURE scale as a validated modification of the DC scale.^8^

Our aim was to understand the impact of a face-to-face consultation on decision making and to determine if prerequisites and the process itself could impact on the final treatment decision, using DC as the outcome measure.

## Methods

### Study population

The cohort consisted of pwMS reviewing DMT options, approached at outpatient clinics across three sites in London (St Mary’s, Charing Cross and Western Eye Hospitals) as part of the Imperial College London Healthcare NHS Trust. The research was conducted between April 16-April 17 as part of the Decisions Of Uncertainty Broaching Treatment in MS (MS-DOUBT) study. The study received ethical approval (REC: 16/LO/0153) and the protocol is available to review. Patients were chosen independent of the lead researcher by neurologists, they had to be aged ≥18 years, have relapsing (RMS) or secondary progressive MS (SPMS) but eligible for DMTs. The patient could be on or off-treatment at the time. ‘Recorded intention to treat’ was a recorded entry in the patient’s medical notes that confirmed or was consistent with an intention recorded in the patient questionnaires. The original cohort who completed the questionnaire in its entirety were re-approached a year later to complete the same questionnaires. All pwMS were offered an MS specialist nurse review prior to starting DMTs, after the study interview. The interview and study questionnaires were referring to the initial consultation with the neurologist. All pwMS starting DMTs are discussed at a multi-disciplinary team meeting following the consultation, where relevant documentation is completed.

### Outcome variables

The primary outcome, DC, was measured using the SURE scale which comprises of four questions answered yes/no: Do you feel SURE about the best choice for you?; Do you know the benefits and risks of each option?; Are you clear about which benefits and risks matter most to you? Do you have enough support and advice to make a choice? Each question of the SURE scale is marked 1 (yes) and 0 (no) and these are then summed (range 0-4: the SURE subscale); lower numbers indicating greater DC (0–3) and 4 representing no DC.

**Figure 1.**
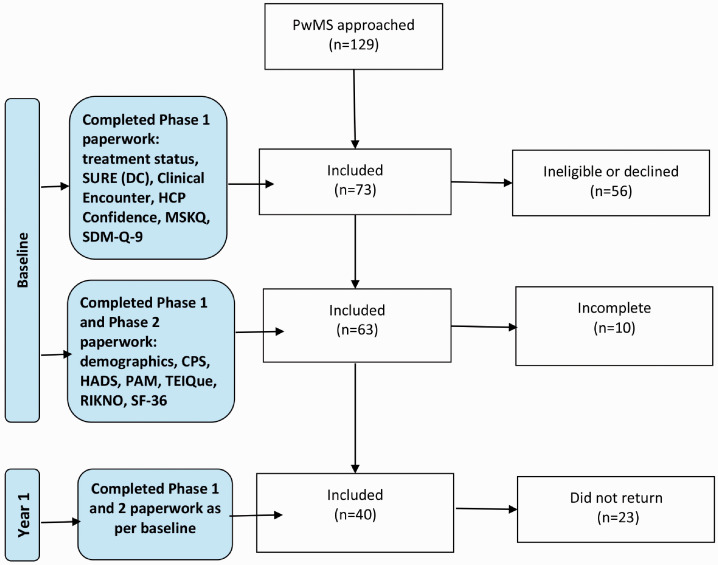
Flow-chart of patient participation per study phase showing questionnaires completed.

The questionnaires were split into Phase 1 and 2 (Figure 1). In the original protocol, the eligibility criteria included a DC status presenting extremes (SURE score = 0 or 4). This would then determine if Phase 2 paperwork was to be completed. The protocol was amended and ethical approval obtained when it was determined that the entry criteria were too stringent and did not reflect the subtleties offered by those with a range of DC, as indicated by the SURE subscale. In addition, treatment status (on or off treatment) and their satisfaction with their treatment status was recorded by the patient.

### Questionnaires

Questionnaire use and administration has been previously described for treatment status,^9^ the Control Preference Scale (CPS)^10^ and the SURE scale.^8^

The Decisional Conflict Gauge (DCG) is a non-validated vertical visual analogue scale (range 0-100)^11^ where the respondent rates his/her DC; higher numbers indicating higher DC

To assess treatment status^9^ PwMS were asked to choose one of four options to categorise them into two groups by treatment status: ‘satisfied’ (on or off treatment but satisfied with current status) or ‘not satisfied’ (on or off treatment and considering options).

We utilised two questions that focused on the clinical encounter.^12^ The first looking at how the ‘clinical encounter’ was communicated in terms of seven questions that were used to generate a communication score. The questions about the HCP included: ‘giving you enough time’, ‘asking about your symptoms’, listening to you’, ‘explaining tests and treatments’, ‘involving you in decisions about your care’, ‘treating you with care and concern’, ‘taking your problems seriously’. The second question, ‘HCP confidence’, addressed how confident the patient was with the HCP seen (definite or partial). These were analysed as per author instructions.^12,13^ Using clinical notes, the cohort was further divided by time of treatment decision: pre-baseline (‘past’), at ‘baseline’ and those who deferred to post-baseline i.e ‘future’ group.

Other questionnaires included: the HADS scale,^14^ the SF-36 questionnaire,^15^ the PAM scale – testing how engaged and ready a person is to make a healthcare decision,^16^ the TEIQue personality questionnaire^17^; the Multiple Sclerosis Knowledge Questionnaire (MSKQ)^18^ and the Risk Knowledge Questionnaire in MS (RIKNO).^19^ The Shared Decision Making Questionnaire was given to pwMS (SDM-Q-9) and HCP (SDM-Q-9-doc).^20^

### Statistical analysis

Analysis of the demographics was performed as previously described.^9^ For the purpose of analysis, DC was reclassified as No DC (0) and DC present (1) but the SURE sub-scale data remained as described previously. Raw values were used for regression analysis unless otherwise stated; some further data conversions were made for T test comparison and are referenced as appropriate: SDM-Q-9^21^, SDM-9-Doc,^21^ MSKQ,^18^ RIKNO^19^ and TEIQue.^17^ The TEIQue was further categorised for the purpose of T-test comparison into values 1-29 (below average), 30-69 (average) and 70-99 (above average). Clinical encounter and HCP confidence^12,13^ were analysed as per author guidance. PAM scores were converted to activation levels.^22^ Only instruments were compared to comparator populations to determine if there were differences. The demographics were not formally analysed due to differences in the way data had been analysed across studies and non-MS and general populations were incorporated where instruments had not been used in an MS population. Data is presented as ratios, percentages and means and standard deviations where appropriate. Statistical analysis was performed using the paired T-test, two-way ANOVA (GraphPad Prism, version 7.02 September 2016: www.graphpad.com). Categorical data was analysed using x2 and Fishers exact test (Vassarstats: www.vassarstats.net accessed 06/08/2019) where appropriate. Modelling the dependence of the scores (DC, SDM) on the covariates was performed using linear and logistic regression models using SPSS (version 22). In logistic regression, covariates were described as odds ratios, reported with 95% confidence intervals and p values testing the null hypothesis of no effect. Graphs were drawn using SPSS, Version 22 and GraphPad Prism (version 7.02 September 2016: www.graphpad.com). All multi-variate analyses were performed using the ‘enter’ method.

### Patient and public involvement

No patient was involved in the creation of the research aims, protocol development or design of the study. The aim is to disseminate the results to participants involved in the research.

## Results

### Demographics and characteristics of the population: MS knowledge; physical and mental health, personality and engagement

**Figure 2.**
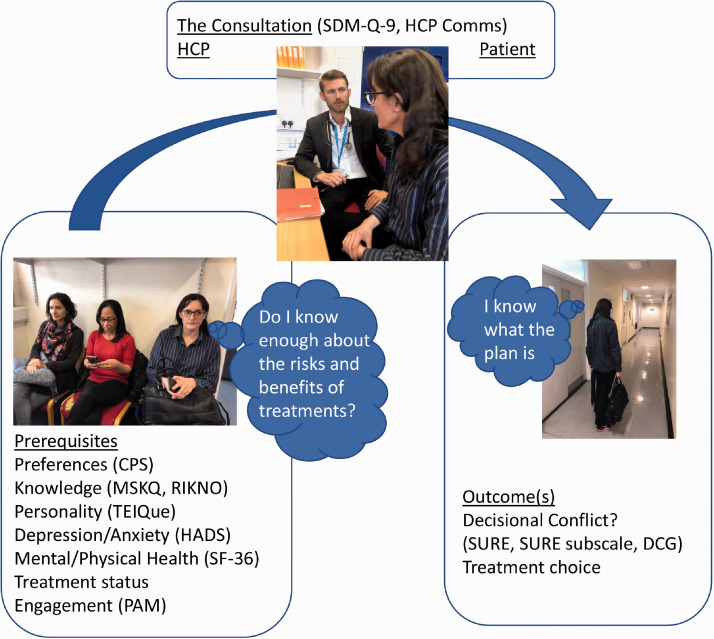
The decisional process model: showing the instruments mapped across the decisional process: prerequisites, the consultation and the outcome measures including treatment choice and DC.

One hundred and twenty nine pwMS were approached immediately after their routine MS specialist consultation. Of these, 73/129 (57%) took part (Figure 1). The stages of the decision process were mapped initially by assessing the patient’s prerequisites, then interrogating the consultation from both the patient’s and HCP’s perspective and finally determining the subsequent outcome of the meeting (Figure 2). The demographics of those who gave informed consent is presented in Table 1. The prerequisites of the decisional process were assessed. It was found that knowledge of MS and treatment risk were positively correlated (n = 60, r^2^=0.261, p < 0.0001), however, knowledge of MS was better than expected for pwMS (See MSKQ, Table 1) but risk knowledge was lower (See RIKNO, Table 1). Comparing the MS group to the general population (GP), there were no differences in mental or physical health (See SF-36, Table 1); though the group had less depression and anxiety than a comparator MS population but more depression than the GP (See HADS, Table 1). Personality and behavioural traits were measured and the only characteristic that differed from the GP was adaptability; meaning this MS group were less adaptable than the GP (See TEIQue, Table 1). As a whole the MS group favoured an active-collaborative role during the consultation (See CPS, Table 1) but they were significantly less engaged than a comparable MS group (See PAM, Table 1).

**Table 1.** Patient demographics and characteristics including prerequisites of the decisional process related to the general population.

	Study population		Comparator population ^(ref)^		
Parameter	N	n (%)	N	n (%)	P value
Relapsing MS	72	68 (94.4%)	51 (28)	34 (66.6%)	<.0001
Gender: female	73	56 (76.7%)	192 (29)	143 (74.4%)	NS
Ethnicity: white	72	59 (81.9%)	5780 (30)	5421 (93.8%)	<0.0002
Without partner	63	40 (63.4%)	1010 (31)	215 (21.2%)	0.004
Employed	66	45 (68.1%)	213 (32)	96 (45.0%)	0.05
Parameter	N	Mean, SD (range)	N	Mean, SD	P value
Age (years)	73	44, 11.34 (21–67)	1387 (33)	40, 5.5	0.002
Disease length (years)	69	7, 7.81 (0–31)	213 (32)	18.9, 11.5	NS
MS knowledge (MSKQ)	73	16.8, 4.2 (3–25)	90 (34)	10.2, 3.2	<0.0001
Risk knowledge (RIKNO)	60	7.1, 3.5 (0–15)	1939 (35)	8.7, 3.5	0.001
Physical status (SF-36)	61	44.0, 10.2 (21–63)	126 (36)	46.0, 10.1	NS
Mental status (SF-36)	61	47.0, 10.7 (20–68)	126 (36)	48.5, 10.1	NS
Anxiety (HADS)	61	7.9, 4.1 (2–20)	144 (37)1792 (38)^a^	8.6, 4.46.14, 3.76	NS0.0009
Depression (HADS)	61	4.7, 3.4 (0–12)	144 (37)1792 (38)^a^	5.9, 3.53.68, 3.07	0.020.02
Overall score (TEIQue)	55	39.0, 24.0 (1–94)	1721(39)^a^542 (40)^a^	50.0, 20.036.7, 12.0	NSNS
Adaptability (TEIQue)	55	28.3, 23.0 (1–98)	1721 (39)^a^	50.0, 20.0	<0.0001
Role preference (CPS)	64	1.6, 0.7 (1–5)	23 (7)	1.8, 0.7	NS
Healthcare management (PAM)	72	55.2, 24.6 (0–100)	199 (16)	63.2, 11.9	0.01

See Ref.^7,16,23–35^

^a^General population.

### High levels of DC is associated with less confidence in healthcare decision-making

During the consultation, it had been made clear a clinical decision about treatment needed to be made unrelated to the study. Of those who took part 39/73 (53%) reported being off treatment at baseline (treatment naïve or off treatment) and 34/73 (47%) were currently on treatment. Of these, 30/34 (88%) were on moderate potency treatment and 4/34 (12%) on high potency treatment. Treatment potency was not associated with differences in DC. Fifty-nine of 73 (81%) were ‘not satisfied’ with their current treatment status. Thirty-nine of 73 (53%) had DC and 32 of the 39 (82%) were also ‘not satisfied’ with their current treatment status (Table 2). A multivariate analysis was performed with all the prerequisites from Table 1. We found that those with less confidence in their healthcare decision-making (PAM) were more likely to have DC using all three measures of DC (n = 72, SURE scale [adjusted R2 0.06, p = 0.02] (Table 3); and independently (n = 72, SURE subscale [adjusted R2 0.04, p = 0.04]; n = 72, DCG [adjusted R2 0.04, p = 0.04]).

**Table 2.** Outcome measures arising from the baseline consultation.

Outcomes by treatment history n (%)	Treatment naïve26 (36%)	Off treatment22 (30%)	On treatment25 (34%)
Not satisfied with treatment, n (% within treatment status)*	22 (37%)	21 (36%)	16 (27%)
Decisional conflict present, n (% within DC)	17 (44%)	10 (26%)	12 (31%)
SURE subscale, mean ± SD	2.46 ± 1.33	2.82 ± 1.56	3.04 ± 1.3
Decisional conflict gauge, mean ± SD	52 ± 26	56 ± 32	42 ± 32

*Treatment status relates to a person’s own evaluation of their satisfaction with treatment – whether they are happy with their treatment status on or off therapy.

**Table 3.** Multivariate analysis of factors associated with the SURE scale measure of DC, SURE subscale, DCG and SDM.

Dependent variable	SURE scale (n = 72)	SURE scale (n = 67)	SURE subscale (n = 72)	SURE subscale (n = 68)	DCG(n = 68)	DCG (n = 68)	SDM (n = 68)
Covariates (prerequisites)	Treatment status, MSKQ, RIKNO,SF36, TEIQue,HADS, CPS, PAM.	Treatment status, MSKQ, RIKNO, SF36, TEIQue, HADS, CPS, PAM.	PAM	PAM	PAM	PAM	Treatment status, MSKQ, RIKNO, SF36, TEIQue, HADS, CPS, PAM.
Covariates (the consultation)		SDM, Clinical encounter		SDM		SDM	Clinical encounter
PAM β, (95%CI lower, upper), p	−.127 (−.233,−.021), 0.020	−.094 (−.184, −.003), 0.04	.312 (.010, .614), 0.04	–	−6.698 (−13.168, −.228), 0.04	–	–
SDMβ, (95%CI lower, upper), p	–	−.012 (−.016, −.008) 0.00	–	.041 (.030, .051), 0.00	–	−.532 (−.816, −.248), 0.00	–
Clinical encounter β, (95%CI lower, upper), p	–	–	–	–	–	–	.985 (.552,1.418), 0.00

The SURE scale - as the primary measure - was used as a dependent variable and run against the covariates described. The SURE sub-scale and DCG were used to support the findings of the SURE scale. The following covariates were used: Treatment Status, MSKQ, RIKNO, SF36 (inc. Physical & Mental), TEIQue (overall score), HADS (Anxiety & Depression), CPS, PAM, SDM, Clinical Encounter or as otherwise stated.

### Optimal shared decision making is associated with less DC

During the consultation, we found 86% had definite confidence in their MS specialist; the remainder reported partial confidence with no one reporting no confidence. Overall, one HCP of a total of five HCPs taking part saw 53/73 (73%) of all patients. This HCP also received a higher HCP satisfaction score over colleagues: 50/53 (94%) with definite confidence in this doctor vs. 13/20 (65%) in the ‘others’ group (p = 0.003). From the patients’ point of view, the overall perceived level of involvement, trust and confidence in the consultation was similar to the GP (Table 4).

**Table 4.** Features of the consultation compared to other populations.

The conversation: features of the consultation					
Parameter	N	Mean, SD (range)	N (ref)	Mean, SD	P value
Clinical encounter	73	89.7, 11.5 (57–100)	7429 (13)^a^	87.5, 17.8	NS
Shared decision making (SDM-Q-9)	68	31.2, 10.6 (3–45)	221 (41)	38.7, 8.5	<0.0001
Shared decision making (SDM-Q-9-Doc)	62	39.2, 6.5 (18–45)87.2, 14.5 (40–100)^b^	10 (42)^c^	80.2, 19.7	NS

Only the patient version of the SDM-Q-9 questionnaire showed a significant difference when compared to an independent MS population. See Ref.^13,36,37^

^a^Non-MS, ^b^This value has been converted again as per the author’s instructions^21^ to enable comparison to non-MS group, ^c^Non-MS group with depression.

The main themes of the consultation that the patient classed as relevant were consideration of ‘Asking about your symptoms’, ‘Listening to you’, ‘Treating you with care & concern’ and ‘Taking your problems seriously’ (Adjusted R^2^ .316, p = 0.000, Table 3). SDM assessment was performed by both patient and doctor after the consultation. The patients’ SDM score was lower than a comparator MS population visiting their general practitioner. The doctors’ SDM assessment reported that the doctor perceived there was significantly more SDM during the consultation than the pwMS identified (for pwMS: SDM-Q-9 69.4, for drs: Q-9-doc 87.21+SD, p = 0.0000; Table 4).

A multivariate analysis was performed for DC, using the same prerequisites but this time including the consultation variables (Table 3). When we added the summed SDM raw scores to the models predicting DC, SDM was a significant factor for DC alongside PAM (n = 67, SURE scale [adjusted R2 0.38, p = 0.000]; and SDM was a standalone driver using SURE subscale (n = 68, [adjusted R2 0.44, p = 0.000]); DCG (n = 68, [adjusted R2 0.16, p = 0.000]). This implied that patients who felt more involved in the process of decision-making also had lower DC.

### Good communication associates with successful SDM

When the SDM score was isolated as a dependent variable and run against the same prerequisites as the DC analysis, the clinical encounter score was the only variable that came out as a significant driver of SDM (Table 3: n = 68, adjusted R2 0.23, p = 0.000). This shows that better communication scores as perceived by the patient during their consultation are associated with successful SDM. There was consensus in 54/72 (75%) when the patient’s treatment choice [e.g start, end, continue, change], was compared to the viewpoint of the doctor’s following consultation, but consensus itself was not associated with DC or SDM measures.

### The final decision arising from the consultation

**Figure 3.**
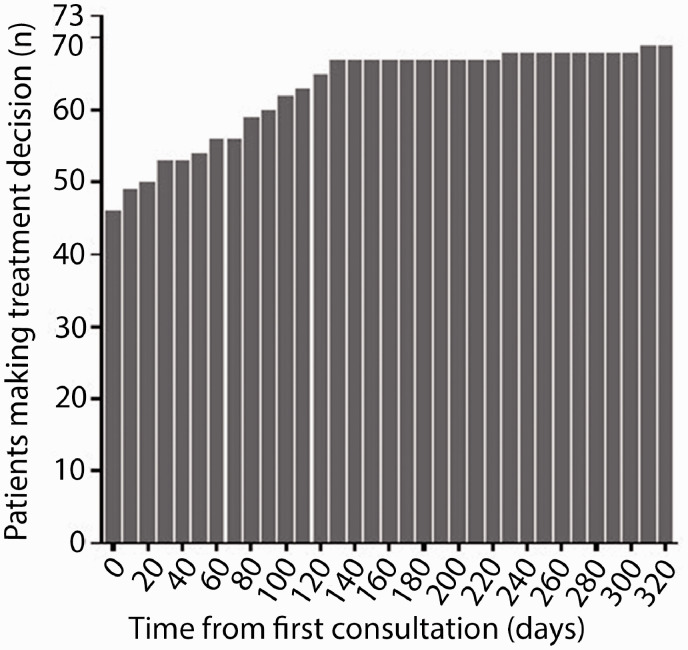
Treatment decision ‘followed through’ as measured by time from consultation. Decisions made after the consultation result in treatment initiation, improved treatment satisfaction and reduced DC.

Overall, 51/73 (70%) of people made their decision at the baseline consultation (41/73, 56%) or reinforced a former decision (10/73, 14%) in the consultation. In the remainder (19/73, 26%), analysis of patient records were used to identify when a decision was made. We found there was a mean of 29 ± 58 days (median of 0 days) from the initial consultation to a recorded intention to treat (Figure 3) with all but 3/73 (4%) following through on the decision by 308 days of the baseline appointment. Of those who made a decision, 11/70 (16%) decided on no treatment, 39/70 (56%) went on to moderate and 20/70 (29%) on to high potency treatment. Having DC at the initial consultation was associated with not starting a treatment (Pearson’s, p = 0.018). We studied those who made a decision before or in the consultation (n = 51, ‘past/baseline’ group) and those after (n = 19, ‘future’ group). We found the ‘future’ group had lower PAM scores though not significant (8/18 [44%, 1 missing] vs 32/51 [63%], p = 0.28) and a trend to have more DC (14/19 (74%) versus ‘past/baseline’ 23/51 (45%), p = 0.057).

Of the past/baseline group, 28/51 (55%) decided on moderate potency treatment, 12/51 (24%) on high potency treatment and 11/51 (22%) had no treatment. Of the future group, 11/19 (58%) decided on moderate potency treatment and 8/19 (42%) decided on high potency treatment.

After one year, we reassessed DC to determine if it had changed. Forty of 73 (55%) responded of which 37 were usable meaning that they completed the DC measures at both time-points. We then compared the future group (n = 10) to the past/baseline group (n = 27). We originally used three measures of DC but by assessing change in DC, we were not able to use the SURE sub-scale measure here. Using the SURE scale 6/10 (60%) of the future group reported their DC resolving compared to 3/24 (13%) of the past/baseline group (Fisher’s p = 0.008). Furthermore the DCG scale also demonstrated a significant improvement, with 8/10 (80%) of the future group reporting a decrease in DC, with the remainder staying the same compared to 9/27 (33%) of the past/baseline group improving, 4/27 (15%) staying the same and 14/27 (52%) showing an increase in DC (p = 0.01). Supporting that this was related to starting treatment, 9/10 (90%) in the future group changed from dissatisfied at baseline to satisfied with their treatment status at year 1 compared to 6/27 (22%) in the baseline/past group (Fisher’s Exact Test 2-sided p = 0.000).

## Discussion

We have shown that in complex decision–making, a well-managed clinical encounter with mutually agreed outcomes as supported by SDM, is associated with less DC in the future, indicating that a ‘good’ decision has been made. In those faced with a complex decision about their MS therapy, dissatisfaction with treatment or not being on it as well as having less confidence in healthcare decision-making is associated with DC. In this context, the face-to-face consultation and optimal SDM appears pivotal to improving outcome in terms of DC with high levels of SDM being associated with lower DC.

By studying when the group made their decision as opposed to actually starting treatment, we found most of the group made their decision prior to or during the consultation with 19/73 (26%) making their decision afterwards and 3/73 (4%) not deciding by a year when followed up. At one year, we found that in those who decided post-consultation, that there were improvements in DC and treatment satisfaction.

We used a range of instruments to map the three stages of decisional process of those deciding about DMTs in MS, with the aim of gaining more insight into how they interact at each stage and impact DC. A key aim here was to understand if DC as an outcome was impacted by the consultation and whether we could use this as a basis in the future to inform the consultation process. Our approach derived from prior work where the failure of a decision aid in diabetes was attributed to missing the doctor/patient interaction.^38^ We tried to ensure that DC was attributed to the DMT decision by framing this within the question^9^ but also we used three measures, two of which were independent measures of DC, to give us further certainty of any findings.

There are often delays commencing DMTs, thus we followed up when the decision to start DMTs was made by checking with the patient and their medical records. When reassessed a year later, again DC was related directly to DMT decision though much may have occurred in the timeframe. For this reason, we used multiple DC measures to verify the results with further support of a link to starting DMTs arising from the fact that the group also had significant improvements in treatment status.

The first stage of decision-making, the prerequisites, are features a patient brings to the consultation. Of the prerequisites, we found that engagement, as measured using the PAM score, is the only consistent feature associated with DC as measured using three different measures. PAM is known to have a real world impact with people who recognise the role of managing their own condition experiencing better healthcare outcomes.^39^

For the consultation, we found that overall the patient had high levels of confidence in the HCP with some HCPs preferred as seen previously.^12,13^ During the consultation, the patient most valued discussion of their symptoms, feeling listened to, being treated with care and concern and that their problems were being taken seriously. Bearing in mind that the consultation principally was about starting therapy, it is interesting that ‘explaining tests and treatment’ and ‘involving you in decisions about your care’ were not significant to patients. This may be giving us a hint as to what is valued by the patient versus the HCPs’ perception of what should be discussed. Reinforcing the importance of this discussion, a good clinical encounter is associated with higher levels of SDM and in turn a high level of SDM perceived by the patient was associated with lower DC. However, again there is evidence of differing perceptions of the consultation, with HCPs’ perception of SDM during the consultation being consistently higher than the patient equivalent.

Allowing a patient to feel they have sufficient time has been found previously to be a key priority for patients as treatment options are time-consuming to communicate.^40^ Here we did not find time itself to be important and the one HCP who received more positive feedback versus colleagues did not spend more time with participants. This suggests that a sense of time can be communicated rather than experienced and may be aligned to the HCP’s own experience and personality. However, though we attempted to align a decision to the consultation itself, decision making is a process with participants being able to decide before, during but also after the consultation in meetings with other HCPs.

Seventy per cent of patients had already made their decision or made it during the baseline meeting. In this group, there was a trend to higher PAM scores and less DC, but the fact that they had made their decision may explain why they were not as concerned about the ‘explaining tests and treatments’ element of the consultation.

In 30%, the decision or not occurred after the meeting and a novel part to this study was that we reviewed the medical notes and followed patients up at a year to ascertain when the decision was made. We found that the decision occurred a mean of 29 days later (range 0-308 days). As far as we can ascertain, there is no data on how long it takes to decide regarding DMTs and 4% had not made a decision by one year. Though only 55% of patients completed the later assessment of those who had made a decision, there was improvement in all DC measures and in treatment satisfaction thus supporting that a successful decision is related to starting treatment.

There are some limitations to the study, there were relatively small participant numbers from one UK NHS Trust and cognitive impairment was not measured though an extensive range of questionnaires needed to be completed. However, this work offers us insight into the process of complex decision-making where multiple HCPs may be involved in the process, but other information sources such as the internet have an increasing influence.^41^ Indeed, patients come to the meeting with a decision made or that they are ready to make a decision. Clearly the consultation with the neurologist is not the only influential factor, though participants were encouraged to reference the consultation with the neurologist. In addition other factors after the consultation including meeting with the specialist nurse could also have an impact, especially in those who decided after the consultation. However, despite this, this work reiterates the status of the clinical encounter^42^ and guides us as to what elements of the consultation are valued; furthermore we have demonstrated how SDM is a vital element for patients. We also find that DC is a useful outcome in this context with the potential to assess the ‘success’ of a clinical encounter. This is important as we have pinpointed areas where HCPs may need to focus to get better outcomes from the consultation.

## Data Availability

Data are available in a public, open access repository. All data relevant to the study are included in the article.
